# Cysteine-Encapsulated Liposome for Investigating Biomolecular Interactions at Lipid Membranes

**DOI:** 10.3390/ijms231810566

**Published:** 2022-09-12

**Authors:** Trang Thi Thuy Nguyen, Seungjoo Haam, Joon-Seo Park, Sang-Wha Lee

**Affiliations:** 1 Department of Chemical and Biological Engineering, Gachon University, Seongnam-si 13120, Korea; 2 Department of Chemical and Biomolecular Engineering, Yonsei University, Seoul 03722, Korea; 3 Department of Chemistry, Eastern University, St. Davids, PA 19087, USA

**Keywords:** liposome, cysteine, gold nanoparticles, colorimetric detection, phospholipase A_2_, Triton X-100

## Abstract

The development of a strategy to investigate interfacial phenomena at lipid membranes is practically useful because most essential biomolecular interactions occur at cell membranes. In this study, a colorimetric method based on cysteine-encapsulated liposomes was examined using gold nanoparticles as a probe to provide a platform to report an enzymatic activity at lipid membranes. The cysteine-encapsulated liposomes were prepared with varying ratios of 1,2-dimyristoyl-sn-glycero-3-phosphocholine (DMPC) and cholesterol through the hydration of lipid films and extrusions in the presence of cysteine. The size, composition, and stability of resulting liposomes were analyzed by scanning electron microscopy (SEM), dynamic light scattering (DLS), nuclear magnetic resonance (NMR) spectroscopy, and UV-vis spectrophotometry. The results showed that the increased cholesterol content improved the stability of liposomes, and the liposomes were formulated with 60 mol % cholesterol for the subsequent experiments. Triton X-100 was tested to disrupt the lipid membranes to release the encapsulated cysteine from the liposomes. Cysteine can induce the aggregation of gold nanoparticles accompanying a color change, and the colorimetric response of gold nanoparticles to the released cysteine was investigated in various media. Except in buffer solutions at around pH 5, the cysteine-encapsulated liposomes showed the color change of gold nanoparticles only after being incubated with Triton X-100. Finally, the cysteine-encapsulated liposomal platform was tested to report the enzymatic activity of phospholipase A_2_ that hydrolyzes phospholipids in the membrane. The hydrolysis of phospholipids triggered the release of cysteine from the liposomes, and the released cysteine was successfully detected by monitoring the distinct red-to-blue color change of gold nanoparticles. The presence of phospholipase A_2_ was also confirmed by the appearance of a peak around 690 nm in the UV-vis spectra, which is caused by the cysteine-induced aggregation of gold nanoparticles. The results demonstrated that the cysteine-encapsulated liposome has the potential to be used to investigate biological interactions occurring at lipid membranes.

## 1. Introduction

Colorimetric sensing strategies have drawn increasing attention due to their simplicity, rapidity, affordability, adaptability, and portability. Colorimetric responses often can be monitored with naked eyes without the use of any advanced instruments. Colorimetric methods have been widely used in chemical or biological sensing, clinical diagnosis, and environmental analysis. Traditionally, organic dyes were investigated extensively as colorimetric probes because some of them can induce color changes through a specific reaction with target analytes, but their applications generally exhibited a limited sensitivity due to the dye’s low extinction coefficient [1]. In contrast, noble metal nanoparticles showing visible color changes depending on their size, shape, and interparticle distance have a great extinction coefficient and they have started to be frequently used as a colorimetric probe to develop a sensitive detection method [2,3,4].

The global outbreak of COVID-19 in recent years showed that the development of a rapid, affordable, and simple testing method is critical to manage such a pandemic by preventing the spread of virus. The most reliable test to detect virus infection is currently a reverse transcription polymerase chain reaction (RT-PCR), which detects the genetic material (RNA) in a virus [5]. However, the RT-PCR test should be performed by trained laboratory personnel in certified clinical laboratories. In addition, the RT-PCR test is time consuming, and it is difficult to use it for point-of-care testing. Nanoparticle-based colorimetric methods can be an alternative outstanding tool to provide quick and accurate clinical testing for a virus, and a few nanoparticle-based strategies have been reported for such testing [5,6,7,8]. The strategies utilized functionalized gold nanoparticles (AuNPs) to target the surface proteins of a virus, detect specific antibodies against a virus, or identify a partial genomic region of a virus. Therefore, those methods can be used to confirm the presence of biological entities including viruses, but their applications are limited in investigating biological activities occurring in a biological system. The study reported here was motivated by an interest in preparing a AuNP-based platform that is able not only to detect targeted biological species but also to monitor biomolecular interactions.

The experimental design of this study was based on the observation that most critical biochemical reactions occur at cell membranes. For example, coronavirus infection is initiated by the attachment of the viral particles to the host cell membrane, which is mediated by the spike (S) proteins on the surface of the virion [9]. Liposome is an artificial vesicle with a spherical shape composed of one or more lipid bilayers, and it provides an excellent method to mimic a cellular membrane [10]. Because liposomes have the aqueous interior and the hydrophobic tails in the bilayer, they can be used as a versatile drug delivery platform by encapsulating and delivering hydrophilic and hydrophobic drugs [10,11,12,13]. Recently, the COVID-19 mRNA vaccines could not be available without the help of liposomes to protect and transport mRNA [10]. Those lipid nanoparticles (LNPs) are composed of phospholipids and cholesterol molecules. Liposomes also have been used as artificial cells to investigate the biological interactions occurring at cell membranes such as membrane fusion [14]. Therefore, the experimental design presented in this study included the use of liposomes to mimic the cell membrane where the biological activities can take place.

A number of colorimetric detection strategies have been developed based on the unique optical properties of AuNPs, and our group has reported a study investigating the cysteine (Cys)-induced aggregation of AuNPs to develop a colorimetric sensor for Cys [15]. The thiol group of Cys bonded to the surface of AuNPs strongly, and the zwitterionic attraction between oppositely charged carboxylate and ammonium groups of Cys led to the aggregation of AuNPs, exhibiting a color change from red to blue [15,16]. The results showed that the presence of Cu^2+^ facilitated the aggregation through the formation of stable complexes between Cu^2+^ and surface-immobilized Cys [15]. The reported system showed a great potential to be used as the AuNP-based sensor to directly detect Cys present in tested samples, but we reasoned that it also may be applicable to the exploration of biological activities that produce or liberate Cys. A past study showed that a Cys-encapsulated liposomal platform composed of sphingomyelin and cholesterol could be used to detect the activity of an enzyme, sphingomyelinase [17]. The strategy can be extended to explore various other kinds of biological interactions when liposomes are prepared from applicable compositions of biological components. By combining the distinct colorimetric response of AuNPs to Cys with the ability of liposomes to encapsulate various molecules, we sought to develop a platform that can be used to investigate the biochemical interactions occurring at cell membranes.

**Scheme 1:**
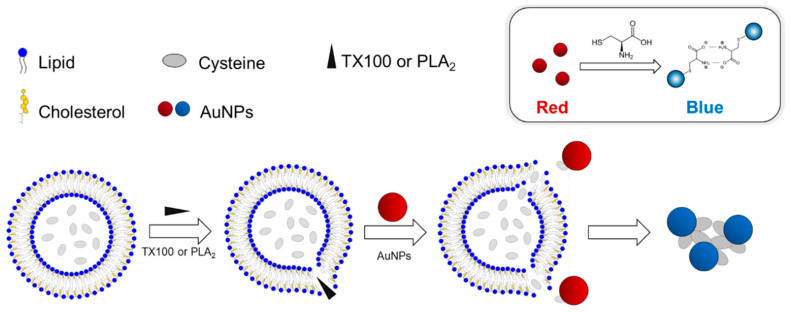
(**Top**) The aggregation and colorimetric response of AuNPs induced by Cys. (**Bottom**) The release of encapsulated Cys by TX100 or PLA_2_ from the liposomes and the subsequent aggregation of AuNPs.

In this report, we describe a new AuNP-based platform including the Cys-encapsulated liposome (CELP) to explore a biomolecular interaction occurring at biological membranes. The CELPs were generated from varying ratios of 1,2-dimyristoyl-sn-glycero-3-phosphocholine (DMPC) and cholesterol (Chol) in the presence of Cys. We reasoned that the release of Cys by a biomolecular interaction at the CELP surface would induce the aggregation of AuNPs, resulting in the color change of the dispersion. To test the hypothesis, the CELP solution was incubated with a nonionic surfactant, Triton X-100 (TX 100), that can disrupt the CELP membrane and release Cys, and then AuNPs were added to test the colorimetric response caused by the Cys. Finally, we tested the system with phospholipase A_2_ (PLA_2_) that can catalyze the hydrolysis of the ester group in DMPC to release Cys from CELP. The schematic description of the aggregation of AuNPs caused by the release of Cys from CELP is shown in Scheme 1.

## 2. Results and Discussion

### 2.1. Preparation and Characterization of Cysteine-Encapsulated Liposomes

Scheme 1 shows the design of the experimental procedure to investigate the molecular interactions at the CELP membrane. When the membrane of the CELP is disrupted by a molecular interaction or a reaction, the encapsulated Cys molecules are released from the liposomes and the released Cys induces the aggregation of AuNPs, leading to the distinctive red-to-blue color change that can be monitored with naked eyes. Past studies showed that the thiol group of Cys can bind to the surface of AuNPs strongly, and the zwitterionic interaction between oppositely charged carboxylate and ammonium groups of Cys attached to adjacent AuNPs can cause the aggregation of AuNPs [15,16]. In the experimental design described in Scheme 1, the CELP should be able to retain Cys molecules over a period of time. Otherwise, the leakage of Cys would cause background signals through the aggregation of AuNPs that cannot be distinguished from the signals induced by the molecular interaction of interest. In order to find the condition to produce stable CELPs, we prepared CELPs with varying ratios of DMPC and cholesterol (Chol). The procedure included the hydration of lipid films in the presence of Cys and subsequent extrusions through a polycarbonate membrane with a pore size of 100 nm. Excessive free Cys molecules were removed by centrifugation before the use of CELPs. The resulting CELPs were characterized by scanning electron microscopy (SEM), dynamic light scattering (DLS), and ^1^H nuclear magnetic resonance (NMR) spectroscopy, and the leakage of Cys from the liposomes were analyzed using Ellman’s reagent.

**Figure 1:**
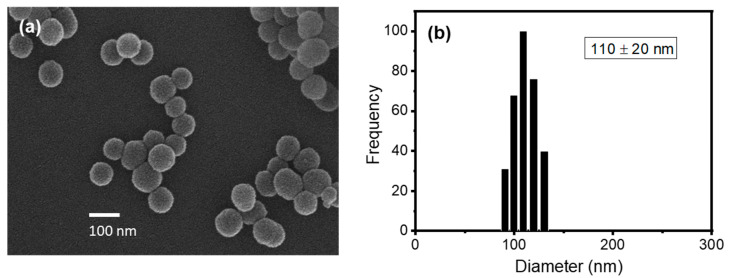
(**a**) SEM image of Cys-encapsulated liposomes and (**b**) size distribution of liposomes measured by DLS. The liposomes were formulated with DMPC:Chol molar ratio of 40:60.

In order to test the activity of phospholipase A_2_ (PLA_2_) as a model biomolecular interaction in the subsequent experiments, liposomes should be prepared with a phospholipid that can be hydrolyzed by the enzyme. Among the potential candidates that are readily available, DMPC was selected as a suitable phospholipid to prepare liposomes for this study. For example, DPPC (1,2-dipalmitoyl-sn-glycero-3-phosphocholine) with two more methylene groups in the chain than DMPC can exist in a non-permeable solid state, which is not appropriate for this study, at room temperature (T_m_ ~ 41 °C). On the other hand, DLPC (1,2-dilauroyl-sn-glycero-3-phosphocholine) with two fewer methylene groups in the chain than DMPC can exist in a leaky liquid state, which might not retain the encapsulated Cys effectively, at room temperature (T_m_ ~ −2 °C). Thus, we reasoned that DMPC, whose transition temperature (T_m_ ~ 24 °C) is close to room temperature, could be a proper candidate because the properties of the resulting liposomes including permeability can be manipulated around room temperature by controlling their composition. The CELPs were formulated from lipid films with DMPC:Chol molar ratios of 60:40 and 40:60. Cholesterol is an essential constituent of most biological membranes and the cholesterol content in a membrane bilayer determines the properties of the membrane [18]. The hydrogen bond between the hydroxyl group in cholesterol with DMPC has been investigated to understand the role of cholesterol in biological membranes [19]. Cholesterol can improve the packing of lipids and affect the fluidity and elasticity. Especially, cholesterol can influence the permeability of water and small chemical species and it is widely used to formulate liposomes for drug delivery [18]. The SEM image in Figure 1a shows the spherical shape of the CELP with a homogeneous size distribution. The DLS analysis confirmed the narrow size distribution of the liposomes, and the average diameter was measured to be 110 ± 20 nm probably due to somewhat aggregation, as shown in Figure 1b. DLS analyses were performed using a Brookhaven light scattering system (BI9000AT/200SM) with a Lexel model 95 argon ion laser at 488 nm.

**Figure 2:**
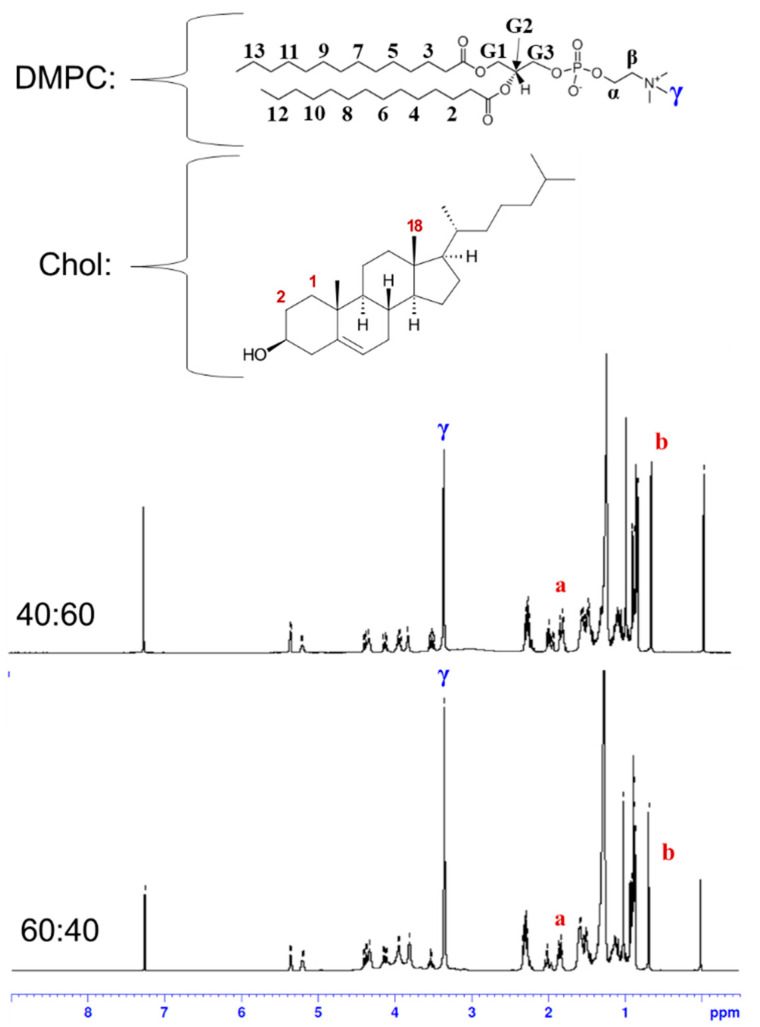
^1^H NMR spectra of liposomes formulated from the lipid films with DMPC:Chol molar ratios of 40:60 and 60:40. The **a** indicates the peak at 1.83 ppm from two protons on carbon 1 and one proton on carbon 2, and the **b** indicates the peak at 0.68 ppm from three protons on carbon 18 in cholesterol.

The liposomes formulated from the DMPC:Chol molar ratios of 40:60 and 60:40 were characterized by ^1^H NMR spectroscopy (Figure 2). Liposomes were prepared through the hydration of lipid films and extrusions as described above, and they were lyophilized and redissolved in CDCl_3_ for the ^1^H NMR measurements. Next, ^1^H NMR spectra were recorded on a Bruker BioSpin Avance III 400 NMR spectrometer operating at 400 MHZ for ^1^H nucleus. The spectra were referenced to tetramethylsilane (TMS) at 0 ppm. The composition of liposomes was determined by comparing the integration of characteristic peaks of DMPC and cholesterol. In the ^1^H NMR spectrum of DMPC, the methyl protons in the trimethyl ammonium group (9H) appeared as a clear singlet at 3.36 ppm, which was not overlapped with other peaks and was labeled as **γ** in the spectra in Figure 2 [20,21]. In the spectrum of cholesterol, two protons on carbon number 1 and one of the protons on carbon number 2 (total 3H) appeared as a multiplet at 1.83 ppm, which was labeled as **a** in Figure 2, and it was well separated from other peaks. According to an NMR study using a deuterated variant of cholesterol, the axial hydrogen on carbon number 2 contributes to the resonance at 1.83 ppm [22]. Another useful peak in the cholesterol spectrum is the singlet at 0.68 ppm from the methyl protons on carbon number 18 (3H), which was labeled as **b** in Figure 2. From the integrals of those peaks, the molar percentages of cholesterol in the liposomes were determined to be 61.9 and 45.8% for the liposomes formulated from the lipid films with DMPC:Chol molar ratios of 40:60 and 60:40, respectively. The initial cholesterol content in the lipid film was matched well with the measured content in the liposome, which indicates that the DMPC bilayer was not saturated with cholesterol yet with these ratios.

### 2.2. Stability of Cysteine-Encapsulated Liposomes

The long-term stability of liposomes is critical in this experimental design because the release of Cys from the liposomes should be occurred only by a specific molecular interaction at the membranes to investigate such an interaction. Therefore, the leakage of Cys from the liposomes was investigated using Ellman’s reagent [23]. Ellman’s reagent (5,5′-dithiobis(2-nitrobenzoic acid) (DTNB)) is often used to quantify free thiol groups in proteins. A free thiol group reacts with DTNB to generate a mixed disulfide and produce a stoichiometric amount of 2-nitro-5-thiobenzoic acid (TNB) that absorbs at 412 nm. Therefore, by measuring the absorbance of TNB, the number of free thiol groups can be determined. The UV-vis spectra of DTNB in the presence of varying concentrations of Cys between 0 μM and 1000 μM (Figure S1a) were measured and the calibration curve was constructed by plotting the absorbance at 418 nm versus the concentration of Cys (Figure S1b). The UV-vis spectra were measured using a Thermo Scientific NanoDrop 2000 UV-vis spectrophotometer at the Smart Materials Research Center for IoT at Gachon University. The results demonstrated a good linearity with the square of the correlation coefficient R^2^ greater than 0.99. The calibration curve was used to determine the concentration of Cys that is leaked from the liposomes formulated from DMPC:Chol molar ratios of 40:60 and 60:40.

The leakage of Cys from the liposomes was monitored with Ellman’s reagent for 150 h at 25 °C. Figure S1c exhibits that the increased cholesterol content in the lipid film generated a more stable liposome. While Cys leaked from the liposome formulated from the DMPC:Chol molar ratio of 40:60 reached the maximum concentration of 390 μM, the liposome formulated from 60:40 showed the plateau of leaked Cys at 540 μM after 75 h. The results suggest that the lipid film with the DMPC:Chol molar ratio of 40:60 generated a more stable liposome that can retain the encapsulated Cys more effectively when compared with that with the DMPC:Chol of 60:40. A number of past studies showed that cholesterol can improve the packing of phospholipids, reducing the permeability of the lipid bilayers and modulating the fluidity, and it can make membranes more rigid at temperatures above the transition temperature [24]. Therefore, the increased cholesterol content can improve the stability of liposomes, making them less leaky. On the other hand, it is also known that the addition of cholesterol decreases the area per molecule in a membrane and significantly increase the membrane thickness, decreasing the internal volume of the liposome [25]. Although none of the data presented in this study provided the estimation of the void volume inside a liposome, it might be possible that the liposome formulated with the higher cholesterol content (60%) has a smaller internal volume and encapsulates less Cys molecules than that formulated with the lower cholesterol content (40%), leading to the lower maximum concentration of released Cys in Figure S1c.

### 2.3. Effect of Concentration of AuNPs on the Colorimetric Response to Released Cys

Our group has investigated the colorimetric response of AuNPs through their aggregation mediated by Cys [15]. The oppositely charged carboxylate and ammonium groups of Cys molecules attached on the surface of adjacent AuNPs caused the aggregation of the particles through zwitterionic attraction, leading to a visible color change from red to blue [15,26]. To use these results to develop the liposome-based sensing strategy described above, it is important to test the visibility of the colorimetric response with naked eyes. If the concentration of AuNPs is too low, the color change cannot be detected visually. On the other hand, if the concentration of AuNPs is too high, the sensitivity of the system can be very low because the visual detection requires an increased amount of Cys. To optimize the concentration of AuNPs to detect Cys, we used Triton X-100 to release Cys from the prepared liposomes. Triton X-100 is a nonionic surfactant that is widely used in biological laboratories to extract proteins and other intracellular materials from cells or permeabilize cell membranes [27]. The polar headgroup of Triton X-100 can disrupt the hydrogen bonding between lipids in a membrane. Thus, Triton X-100 has the ability to penetrate lipid bilayers and destroy the integrity of a membrane. We used Triton X-100 to release Cys from the Cys-encapsulated liposomes and tested the resulting visibility of the colorimetric response of AuNPs to the released Cys.

In order to examine the visibility, AuNPs were prepared by the citrate reduction method. The TEM image and DLS result in Figure S2a,b showed the monodisperse distribution of spherical AuNPs with 19.1 ± 0.3 nm diameter. In Figure S2c, the XRD pattern of the AuNPs showed four characteristic peaks at 2θ = 37.84°, 43.88°, 64.46°, and 77.42° corresponding to (111), (200), (220), and (311) planes of a face center cubic (fcc) lattice of gold, respectively [28]. The UV-vis spectra exhibited that the cetyltrimethylammonium bromide (CTAB)-capped AuNPs remained stable in the various buffer systems examined in this study, but citrate-stabilized AuNPs showed a red-shift from 520 nm to ~700 nm in a couple of buffer systems, indicating the aggregation of AuNPs (Figure S3). Therefore, the citrate-stabilized AuNPs were stirred with CTAB for 2 h to form a CTAB layer on the surface of the AuNPs before use [29].

**Figure 3:**
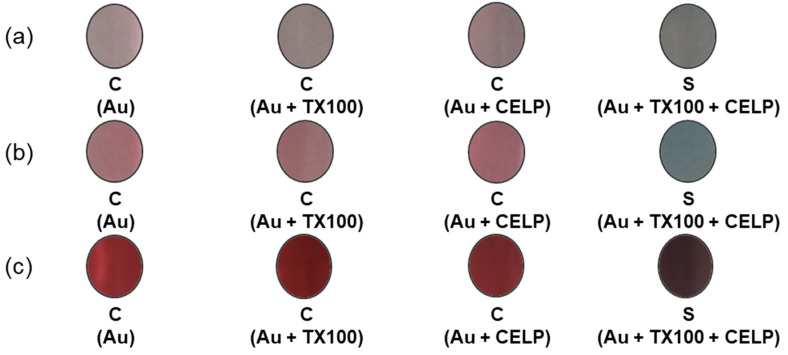
Colorimetric responses of AuNPs at different concentrations: (**a**) 0.06 mM, (**b**), 0.12 mM, (**c**) 0.24 mM.

For the visibility test, the aggregation of AuNPs at three different concentrations (0.06 mM, 0.12 mM, and 0.24 mM) was tested with cysteine-encapsulated liposomes and Triton X-100 (Au + TX100 + CELP) in HPLC grade water at pH 7. For comparison, the aggregation of AuNPs was also investigated under the following conditions: (1) in the absence of Triton X-100 and the liposomes (Au), (2) in the absence of the liposomes (Au + TX100), and (3) in the absence of Triton X-100 (Au + CELP). After the 1 h incubation of all testing aqueous solutions (just water, TX100, CELP, and TX100 + CELP), AuNPs were added, and the color changes were recorded after 10 min of the addition. According to Figure 3, the samples without Triton X-100 and/or liposomes (Au, Au + TX100, and Au + CELP) maintained the original red color of AuNPs, but the sample with the liposomes treated with Triton X-100 (Au + TX100 + CELP) exhibited visual color transitions.

The UV-vis spectra of Au, Au + TX100, and Au + CELP samples at all three different concentrations of AuNPs showed the characteristic surface plasmon resonance peak at 520 nm (Figure S4). However, when the liposomes were incubated with Triton X-100 before the addition of AuNPs (Au + TX100 + CELP), the peak at 520 nm diminished and the secondary peak at 670 nm appeared noticeably, indicating the aggregation of AuNPs. For the samples with the lowest concentration of AuNPs (0.06 mM), the peak at 520 nm was almost flattened and the peak at 670 nm increased significantly, showing the most significant change in the UV-vis spectra upon the treatment of liposomes with Triton X-100 (Figure S4a). Nevertheless, the series of the samples with 0.06 mM AuNPs did not exhibit a clear color change between Au + TX100 + CELP and others (Figure 3a), probably because of insufficient AuNPs for visualization. On the contrary, the samples with the highest concentration of AuNPs (0.24 mM) showed dark colors and a color change for Au + TX100 + CELP (Figure 3c) that can be monitored with naked eyes; although, the UV-vis spectra presented the least pronounced change among all three examined concentrations of AuNPs (Figure S4c). As we hypothesized above, if the concentration of AuNPs is too high, the amount of released Cys could not be enough to induce the aggregation of all existing AuNPs, resulting in an incomplete disappearance of the peak at 520 nm regardless of the color change that can be recognized. The peak at 520 nm was still prominent and larger than the peak at 670 nm (Figure S4c). Additionally, the colors of the samples were also very dark, and it was not easy to perceive the color change (Figure 3c). In comparison with the samples with the lowest and highest concentrations of AuNPs, those with 0.12 mM AuNPs demonstrated the modest change in the UV-vis spectra upon the treatment of liposomes with Triton X-100 (Figure S4b) but still a noticeable color change that can be easily recognized without the support of any instruments (Figure 3b). Thus, in order to achieve distinctive visibility and good sensitivity, the AuNPs at 0.12 mM were used for the subsequent experiments, unless otherwise specified.

### 2.4. Effect of pH on the Colorimetric Response

We have tested the effect of pH on the colorimetric response of the cysteine-encapsulated liposome system. For the test, the samples were prepared in HPLC grade water (pH 7), phosphate buffered saline (PBS) (pH 7.4), an aqueous HCl solution (pH 5), and PBS (pH 5.2). As in the visibility test, Triton X-100 was used to induce the release of Cys from the liposomes by the interaction at the membrane. As a nonionic surfactant composed of poly(ethylene glycol) moiety, the interfacial activity of Triton X-100 is not affected by acidic pH as low as 1.5 [30]. Therefore, Triton X-100 can disrupt the membranes of liposomes effectively over the range we examined in this study. The liposomes were formulated from the lipid films with the DMPC:Chol molar ratio of 40:60, and the concentration of AuNPs was adjusted to be 0.12 mM for the optimum visibility.

**Figure 4:**
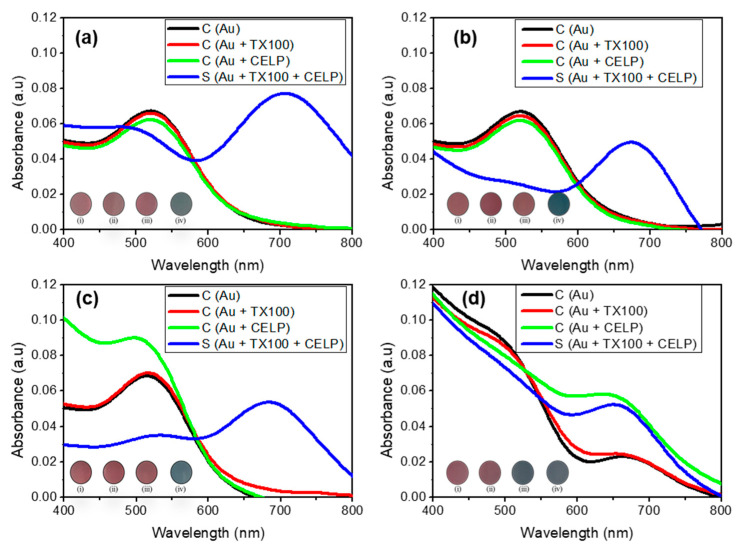
The UV–vis spectra of 0.12 mM AuNPs in various conditions and media: neutral pH (**a**), phosphate buffered saline (pH 7.4) (**b**), HCl-adjusted water (pH 5) (**c**), and phosphate buffer saline (pH 5.2) (**d**). Insets show the corresponding photographs of AuNPs: only AuNPs (i); AuNPs with TX100 (ii); AuNPs with CELP (iii); AuNPs with TX100 and CELP (iv).

Figure 4 shows the UV-vis spectra of Au, Au + TX100, Au + CELP, and Au + TX100 + CELP in various media. All spectra in the absence of the liposomes (Au and Au + TX100) showed only one peak at 520 nm regardless of the media and pH, which indicates that Triton X-100 or the medium itself does not induce the aggregation of AuNPs under the given conditions. In contrast, when cysteine-encapsulated liposomes were incubated with Triton X-100 before the addition of AuNPs (Au + TX100 + CELP), the peak at 520 nm decreased and the peak around 650–700 nm increased significantly in all tested media, leading to a distinct red-to-blue color change. Here, we estimated the aggregation degree of AuNPs by the absorbance ratio (A_670/520_) determined at 670 and 520 nm in the UV-vis spectra. The results suggest the color change might occur from the aggregation of AuNPs by the Cys released from the liposomes through the disruption of their membranes by Triton X-100. To support this proposition, we tested the AuNPs in the presence of cysteine-encapsulated liposomes without incubating with Triton X-100 (Au + CELP). In HPLC grade water (pH 7), PBS (pH 7.4), and HCl solution (pH 5), Au + CELP did not show the peak at 650–700 nm, indicating stable encapsulation of Cys in the liposomes; however, in PBS (pH 5.2), the Au + CELP presented the sign of aggregation with the appearance of the peak around 650–700 nm in the UV-vis spectrum (Figure 4d) and with the red-to-blue color transition. The change of Au + CELP in PBS (pH 5.2) suggests that Cys can be leaked from the liposomes even without contacting with Triton X-100 in the buffer medium. We reasoned that the relatively high ionic strength in PBS can reduce the electrostatic repulsion between liposomes and facilitate the aggregation and fusion of cysteine-encapsulated liposomes, accompanying with the leakage of Cys [31]. However, the Au + CELP in PBS (pH 7.4) did not show such a change (Figure 4b) and it might be possible that the change of Au + CELP was caused by the synergic effect of ionic strength and pH.

In order to test the stability of cysteine-encapsulated liposomes in acidic buffer solutions, the colorimetric responses of our sensor system were further investigated in sodium acetate buffer (SAB) at pH 5.5 and citrate phosphate buffer (CPB) at pH 5.0. As shown in Figure S5, the cysteine-encapsulated liposomes in SAB and CPB exhibited almost the same behavior as those in PBS at pH 5.2 shown above (Figure 4d). In the absence of the cysteine-encapsulated liposomes, the Au and Au + TX100 did not show any color changes in the tested buffer solutions. However, the Au + CELP showed a clear sign of aggregation of AuNPs with the appearance of the peak around 650–700 nm in the UV-vis spectra (Figure S5) and the accompanying red-to-blue color change in both SAB (pH 5.5) and CPB (pH 5.0) even without Triton X-100. This observation might be explained by two possible reasons. First, the AuNPs might be unstable in the presence of liposomes and the liposomes can induce the aggregation of AuNPs. Second, the cysteine-encapsulated liposomes have a limited stability in both acidic buffer solutions as well as in PBS (pH 5.2) and the encapsulated cysteine might leak from the liposomes, resulting in the cysteine-mediated aggregation of AuNPs. To test the first possibility, AuNPs were incubated with liposomes lacking cysteine in various buffer solutions. Figure S6 did not show a distinct peak appearance around 650–700 nm; thus, we concluded the color change of Au + CELP is mainly caused by cysteine leaked from the liposomes in acidic buffer solutions rather than directly by liposomes.

### 2.5. Application of the Sensing Strategy to Investigate an Enzymatic Activity

The results above showed the potential of this cysteine-encapsulated liposome-based system to investigate biomolecular interactions occurring at biological membranes. We tested phospholipase A_2_ (PLA_2_) from the porcine pancreas as a model enzyme to demonstrate the feasibility of the system to report an enzymatic activity occurring at the membrane interface. The Ca^2+^-regulated hydrolysis of phospholipids by PLA_2_ is stereoselective and only the sn-2 ester bond of L-phospholipids can be hydrolyzed to a free fatty acid and 1-lysophospholipid [32]. It has been reported that the hydrolysis products of an L-phospholipid by PLA_2_ desorb from the lipid membrane [33], and we hypothesized that the enzymatic reaction can result in the release of encapsulated cysteine from the liposomes. The activity of PLA_2_ was tested in HPLC water (pH 7) and PBS (pH 7.4).

**Figure 5:**
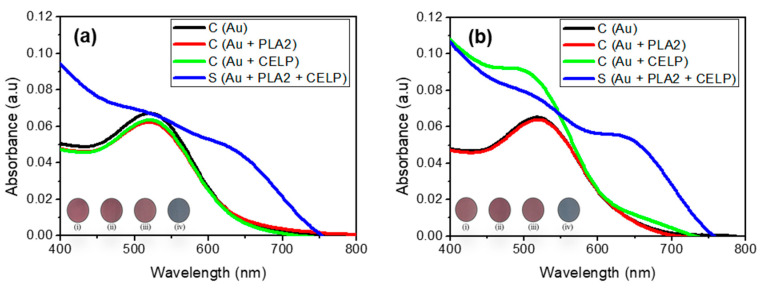
The UV–vis spectra of AuNPs (0.12 mM) under activity of PLA_2_ in HPLC water at neutral pH 7.0 (**a**), and phosphate buffered saline (pH 7.4) (**b**). Insets show the corresponding photographs of AuNPs: (i) AuNPs, (ii) AuNPs with PLA_2_, (iii) AuNPs with CELP, (iv) AuNPs with both CELP and PLA_2_.

Figure 5 shows the UV-vis spectra of Au, Au + PLA_2_, Au + CELP, and Au + PLA_2_ + CELP tested in HPLC water (pH 7) and PBS (pH 7.4). All spectra of control samples (Au, Au + PLA_2_, and Au + CELP) showed only one peak at around 520 nm in both media, which indicates that only PLA_2_ or CELP do not induce the aggregation of AuNPs under the given conditions. The Au + CELP in PBS at pH 7.4 showed a slight increase in absorbance at around 690 nm but no visible color change was observed (iii in the inset in Figure 5b). The results show that the cysteine-encapsulated liposomes are reasonably stable at a neutral pH and the leakage of cysteine is not detectable. In contrast, when both PLA_2_ and cysteine-encapsulated liposomes were added to gold nanoparticles (Au + PLA_2_ + CELP), a new peak around 650–700 nm increased significantly in both media, leading to a distinct red-to-blue color change. Such changes suggest that PLA_2_ induces the leak of cysteine from the liposomes and the released cysteine can facilitate the aggregation of gold nanoparticles. As explained above, it is known that the hydrolysis products from L-phospholipids by PLA_2_ can desorb from the liposomes, resulting in the release of cysteine. Overall, the results from the test of PLA_2_ demonstrated that the cysteine-encapsulated liposomes can be successfully used to investigate biological interactions occurring at cell membranes.

### 2.6. Summary of Colorimetric Responses of AuNPs by Cysteine-Encapsulated Liposomes with Triton X-100 or PLA_2_

**Figure 6:**
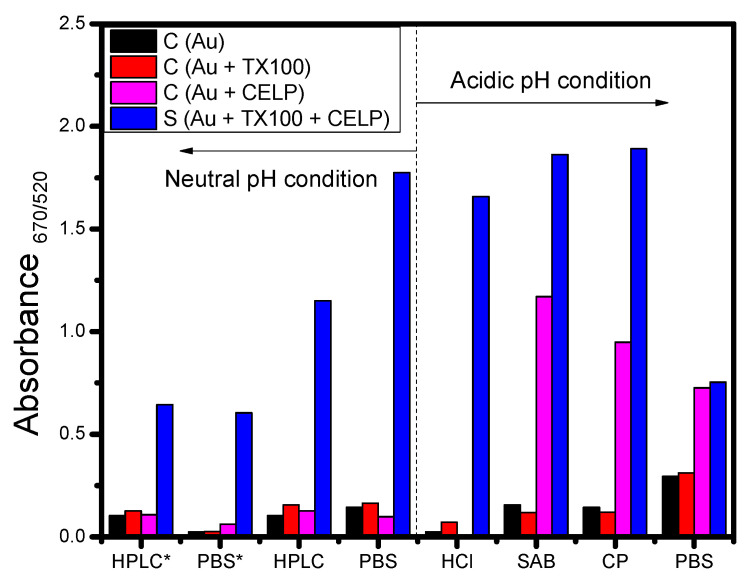
Absorbance ratios of 3 control samples (AuNPs, AuNPs with TX100 or PLA_2_, and AuNPs with liposome) and the sample including AuNPs, TX100 (or PLA_2_), and liposomes after incubation for 1 h in various buffer media. * indicates the use PLA_2_ instead of TX100.

From the UV-vis spectra data shown in Figure 4 and Figure 5, the absorbance ratio measured at 670 nm and 520 nm was calculated and used to estimate the degree of aggregation of gold nanoparticles. Figure 6 shows the absorbance ratio (A670/520) for Au, Au + TX100, Au + CELP, and Au + TX100 + CELP in various media and the asterisk indicates the data obtained from PLA_2_ instead of Triton X-100. In Figure 6, Au and Au + TX100 (or Au + PLA_2_) showed the absorbance ratio less than 0.3 in all tested media without a noticeable sign of aggregation of nanoparticles. However, Au + CELP showed significantly increased absorbance ratios in acidic buffer media, demonstrating the possible leak of cysteine from the liposomes, and resulting aggregation of nanoparticles. The results might suggest the limited stability of cysteine-encapsulated liposomes in acidic buffer solutions and the use of the liposomes as a colorimetric sensor under such conditions should be applied more cautiously. In contrast, at neutral pH regardless of media, Au, Au + TX100 (or Au + PLA_2_), and Au + CELP did not show any perceptible increase in the absorbance ratio (A670/520), and the presence of Triton X-100 or PLA_2_ could be selectively confirmed by monitoring the UV-vis spectra or observing visible color change.

## 3. Methods and Materials

### 3.1. Chemicals

First, 1,2-dimyristoyl-sn-glycero-3-phosphocholine (DMPC, powder) was purchased from Avanti Polar Lipids (Alabaster, AL, USA). Tetrachloroauric (III) acid (HAuCl_4_·3H_2_O, 99.9%), trisodium citrate (Na_3_C_6_H_5_O_7_, meets USP testing specifications), L-cysteine hydrochloride (≥98%, C_3_H_7_NO_2_S·HCl), 5,5′-dithiobis(2-nitrobenzoic acid) (≥98%, bioreagent, suitable for determination of sulfhydryl groups), cholesterol (≥99%, Sigma grade), Triton^TM^ X-100 (laboratory grade), phospholipase A_2_ (from porcine pancreas), cetyltrimethylammonium bromide (CTAB), methanol, chloroform, phosphate buffer saline (pH 7), phosphate buffer saline (pH 5.2), sodium acetate buffer (pH 5.5), citrate phosphate buffer (pH 5.0), hydrochloric acid (36%, HCl), and HPLC grade water were purchased from Sigma Chemical Co. (Saint Louis, MO, USA) All reagents were analytically pure. Glassware were cleaned with aqua regia (HCl:HNO_3_ = 3:1 vol/vol) and subsequently rinsed thoroughly with deionized water more than three times before use.

### 3.2. Preparation of Liposomes

To synthesize the liposomes, 1,2-dimyristoyl-sn-glycerol-3-phosphocholine (DMPC) and cholesterol (Chol) were dissolved in methanol and chloroform, respectively, to have the same concentration (10 mg/mL). The solutions were mixed such that the molar ratio of DMPC to Chol was 60:40 (0.2 mL and 0.18 mL) or 40:60 (0.3 mL and 0.12 mL) in a 50 mL round-bottom flask, respectively. Then, the solvents were evaporated for 5 min by a rotary evaporator to form a thin film. After the residual solvent was gradually removed under vacuum for 12 h, 1 mL of Cys solution was prepared in phosphate-buffered saline (PBS, pH 7.4) and added into the lipid film at room temperature. The flask was agitated on a vortex mixer until the lipid film is dispersed in the solution. To obtain a uniform distribution of particles, the suspension was sonicated for 30 min at 30 °C and the solution was passed through a mini-extruder (Avanti Polar Lipids) that was equipped with a polycarbonate membrane filter with a pore size of 100 nm 31 times. In the final step, free Cys was removed from liposomes by using a microcentrifugation more than three times for 3 min at 10,000 rpm. Liposomes were stored at −20 °C prior to further characterization.

### 3.3. Synthesis of AuNPs

AuNPs were synthesized by a citrate reduction process. In brief, 50 mL of tetrachloroauric acid (HAuCl_4_) (0.01 wt. %) in a 250 mL round-bottom flask was heated up to 100 °C under continuous stirring. To the boiling solution, 3 mL of 1.0 wt. % sodium citrate solution was quickly poured and the solution color changed into red after 8 min. After boiling for an additional 10 min, the flask was moved to a stirrer at room temperature and the solution was stirred for 2 h. The resulting AuNPs were stored in a refrigerator at 4 °C before further experiments. To form the CTAB layer on the AuNPs, 2 mL of the citrate-stabilized AuNPs were mixed with 2 mL of 5 mM CTAB solution under constant stirring for 2 h.

### 3.4. Determination of Cys Concentration

The concentration of free primary sulfhydryl groups of Cys was determined by using 5,5′-dithiobis(2-nitrobenzoic acid) (DTNB) in neutral and alkaline conditions. First, a series of Cys solutions (0 μM, 200 μM, 400 μM, 600 μM, 800 μM, and 1000 μM) were prepared in deionized (DI) water. Each prepared solution (0.4 mL) was added into 0.01 M DTNB solution (0.2 mL), which was diluted with 0.3 M Tris-HCl buffer at pH 8.0. Then, the Nanodrop spectrophotometer was used to measure the UV-vis spectra of the reacted solutions after 5 min. To determine the Cys concentration leaked from the liposomes, the same procedure was performed with the 4:2 volume ratio of liposome and DTNB solutions. The analytical instruments used in this study are described in the Supporting Information.

## 4. Conclusions

In this study, the cysteine-encapsulated liposomes were prepared and examined to provide a platform to investigate biomolecular interactions occurring at cell membranes. The liposomes were formulated from lipid films with varying molar ratios of DMPC and cholesterol (60:40 and 40:60) through hydration and subsequent extrusions in the presence of cysteine. The SEM and DLS results showed the formation of a homogeneous size distribution of spherical liposomes, and the average diameter was estimated to be 110 ± 20 nm. The ^1^H NMR spectra confirmed that the initial DMPC:Chol molar ratio in the lipid film was maintained well in the resulting liposome. For example, the molar percentage of cholesterol in the liposome was determined to be 61.9% when formulated from the lipid film containing 60 mol % cholesterol. The leakage of cysteine from the liposomes was tested with Ellman’s reagent and UV-vis spectrophotometry, and the results suggest that an increased cholesterol content may decrease the leak of encapsulated cysteine. Specifically, the liposome formulated with the DMPC:Chol molar ratio of 40:60 retained encapsulated cysteine molecules more effectively than that formulated with DMPC:Chol of 60:40 over an extended period of time (150 h). A nonionic surfactant Triton X-100 was used to disrupt the liposome membranes to release the encapsulated cysteine, and the released cysteine was detected by the distinct color change of gold nanoparticles through cysteine-mediated aggregation. The visibility of the color change was examined with three different concentrations of AuNPs (0.06, 0.12, and 0.24 mM) and among the tested concentrations, 0.12 mM AuNPs exhibited the most distinct color change that can be easily monitored with naked eyes upon the addition of Triton X-100. The AuNPs with the highest concentration (0.24 mM) showed too dark colors and it was difficult to perceive the color change. The samples were tested in HPLC grade water at pH 7, PBS at pH 7.4, an aqueous HCl solution at pH 5, and PBS at pH 5.2. Except in PBS at pH 5.2, the color change of the solutions was observed only after the cysteine-encapsulated liposomes were incubated with Triton X-100. However, in PBS at pH 5.2, the solution of liposomes including gold nanoparticles showed the red-to-blue color change even in the absence of Triton X-100. The same nonspecific color change was also observed in SAB at pH 5.5 and CPB at pH 5.0. Since such a change was not detected in PBS at pH 7.4 and the HCl solution at pH 5, it might be caused by the synergic effect of a high ionic strength and an acidic pH. Finally, the liposomal platform was tested if it can be used to investigate an enzymatic activity occurring at lipid membranes using PLA_2_ as a model enzyme. PLA_2_ can catalyze the hydrolysis of the ester group in phospholipids in the liposome, resulting in the release of encapsulated cysteine. The cysteine-encapsulated liposomes exhibited the red-to-blue color change caused by the released cysteine-induced aggregation of AuNPs only when treated with PLA_2_ in HPLC grade water at pH 7 and PBS at pH 7.4. The activity of PLA_2_ could also be successfully confirmed by the appearance of a peak around 690 nm in the UV-vis spectra, indicating the aggregation of AuNPs. Although the investigated strategy using the cysteine-encapsulated liposome was tested only with PLA_2_ as a model enzyme, the results suggest that this liposome-based platform can be used as a simple but useful tool to investigate biomolecular interactions at lipid membranes when the liposome is prepared with an appropriate composition of specific lipids. Given that many critical biomolecular processes take place at cell membranes, we believe that this approach will find various applications in medical practice.

## Data Availability

Not applicable.

## Supplementary Materials

The following supporting information can be downloaded at: https://www.mdpi.com/article/10.3390/ijms231810566/s1.
