# Supplementary material for: Cysteine-Encapsulated Liposome for Investigating Biomolecular Interactions at Lipid Membranes
Source: Int J Mol Sci. 2022 Sep 12;23(18):10566. doi: 10.3390/ijms231810566 (PMC9500635; doi:10.3390/ijms231810566)
Supplement: Supplementary file 1 [file ijms-23-10566-s001.zip › ijms-1891004-supplementary.pdf]

Supporting Information

Instrumental Analysis

The particle size distribution of samples was determined by dynamic light scattering (DLS). The size and surface morphology of liposomes and gold nanoparticles (AuNPs) were characterized by scanning electron microscopy (SEM; Hitachi S-4700 FE-SEM; Japan) and transmission electron microscopy (TEM; 80kV, Hitachi H-7600, Japan), respectively. The crystalline structure of AuNPs was measured by X-ray diffraction (XRD; Rigaku Rint 2200 Series, Rigaku, Tokyo, Japan) using Cu Kα1 radiation of wavelength λ = 1.5406 Å, at 40 kV voltage and 30 mA current with the continuous-scanning 2ɵ mode. The composition of liposomes was characterized by proton nuclear magnetic resonance (^{1}H NMR; 400 MHz, Agilent Technologies, Santa Clara, CA).

Generally, AuNPs prepared by citrate reduction method showed a negative zeta potential of -26.51 ± 0.99 mV. Fig. S2 showed the UV-vis spectra of citrate-capped AuNPs (a) and CTAB-coated AuNPs (b) in various conditions. In water, PBS buffer, and HCl solution of AuNPs/citrate, spectra exhibited a sharp peak at 520 nm, however, the peak was shifted from 520 nm to 700 nm in CPB and SAB. The zeta potential of the CTAB-stabilized AuNPs was measured as +8.36 ± 0.23 mV. A layer of positively charged CTAB can be formed on the negatively charged surface of AuNPs [1]. In the spectra of CTAB-capped AuNPs in various buffers, the peak at 520 nm remained stable. The surface energy of AuNPs was decreased by CTAB surfactant layer and CTAB-capped AuNPs was used for this investigation.

1. Li, R., Wang, Z., Gu, X., Chen, C., Zhang, Y., and Hu, D.: 'Study on the assembly structure variation of cetyltrimethylammonium bromide on the surface of gold nanoparticles', ACS Omega, 2020, 5, (10), pp. 4943-4952.

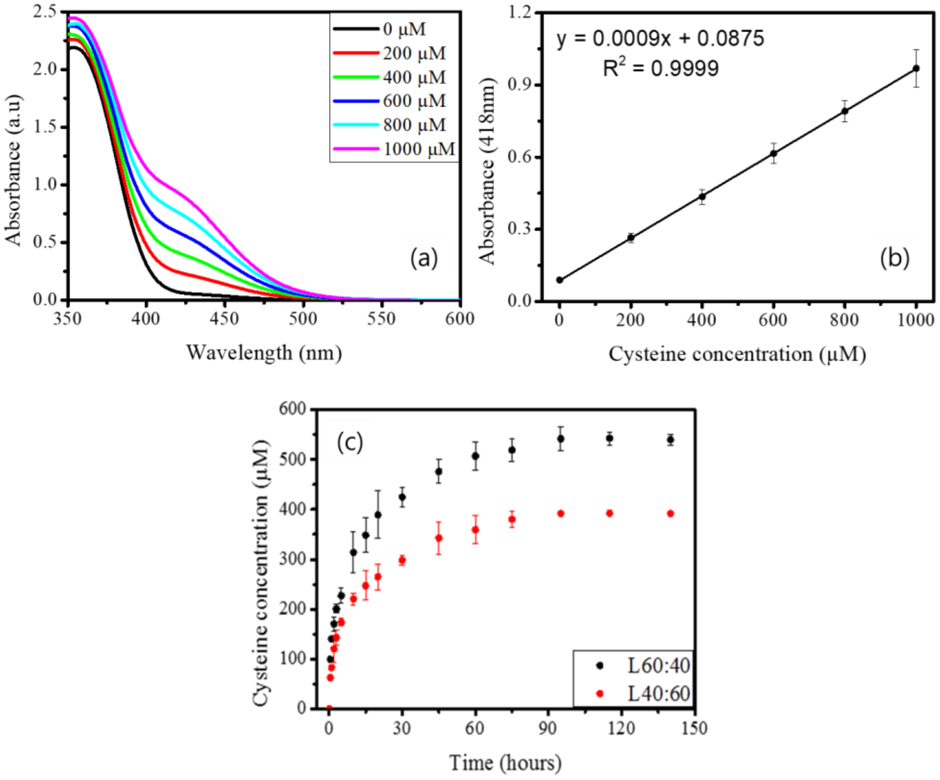

Figure S1. (a) The UV-vis spectra of DTNB in the presence of varying concentrations of Cys (0 μM, 200 μM, 400 μM, 600 μM, 800 μM, 1000 μM) and (b) corresponding calibration curve at 418 nm. (c) Leakage of Cys from the liposomes formulated from DMPC:Chol 40:60 and 60:40 mol % lipid films over time.

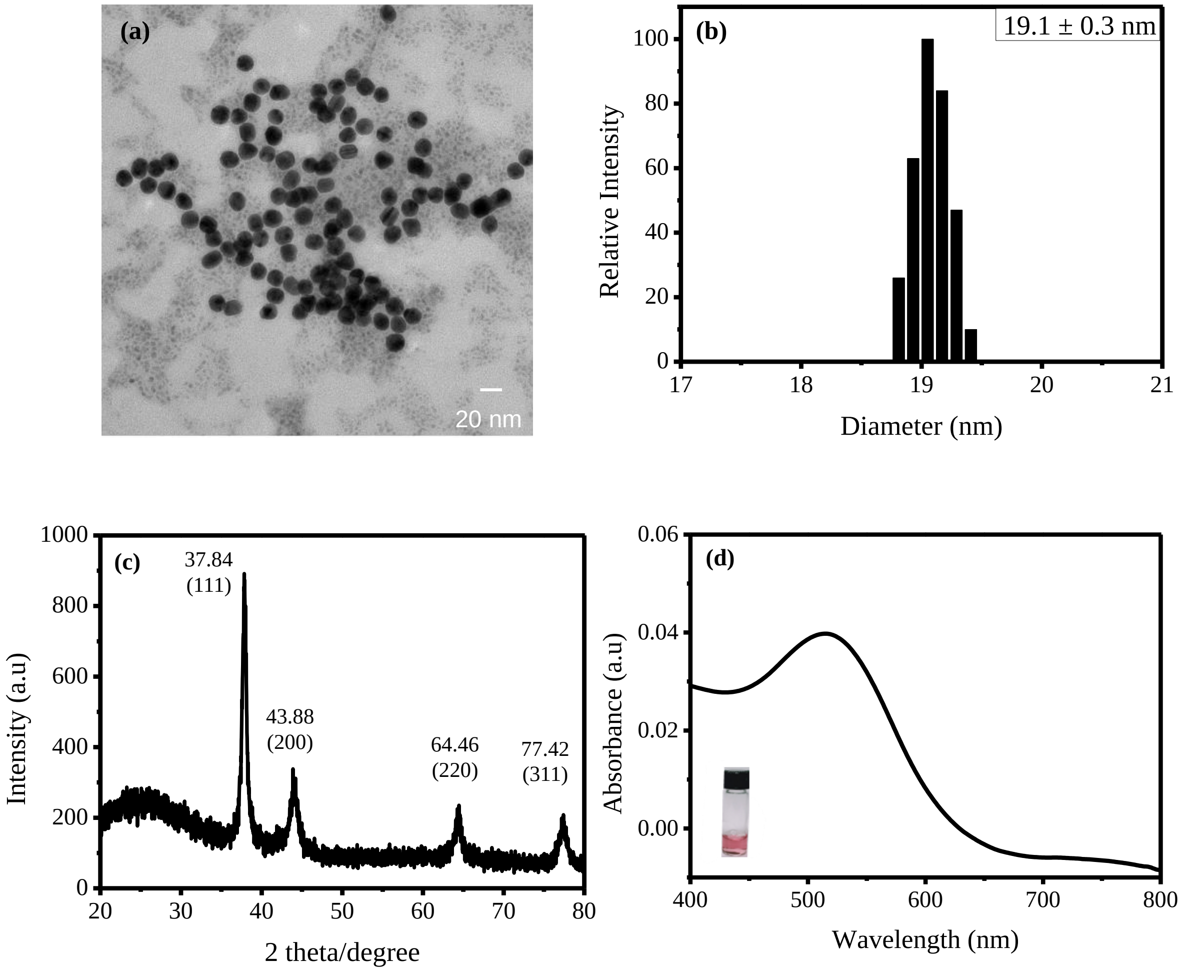

Figure S2. (a) TEM images, (b) size distribution determined by DLS, and (c) XRD pattern of AuNPs prepared by citrate reduction method. (d) UV-vis spectrum of the CTAB-capped AuNPs.

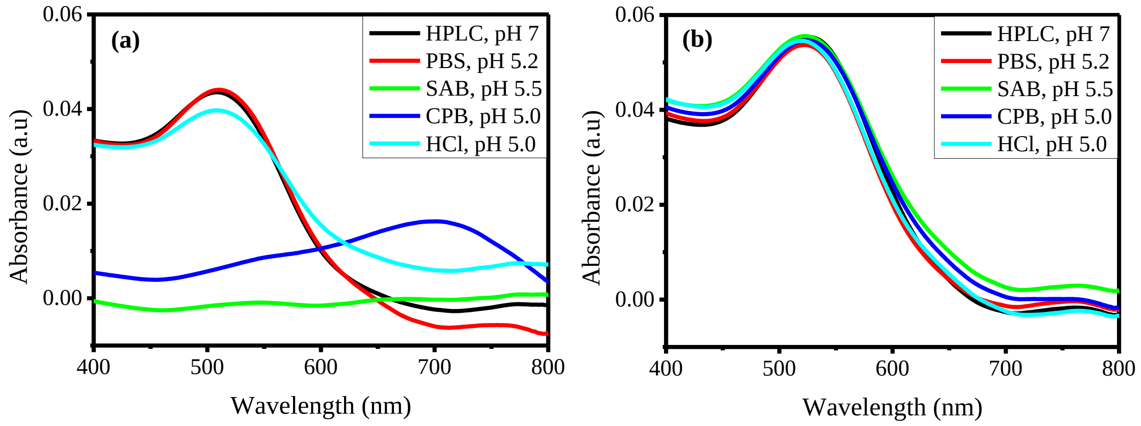

Figure S3. The UV-Vis spectra of (a) citrate-capped AuNPs and (b) CTAB-capped AuNPs in various buffers.

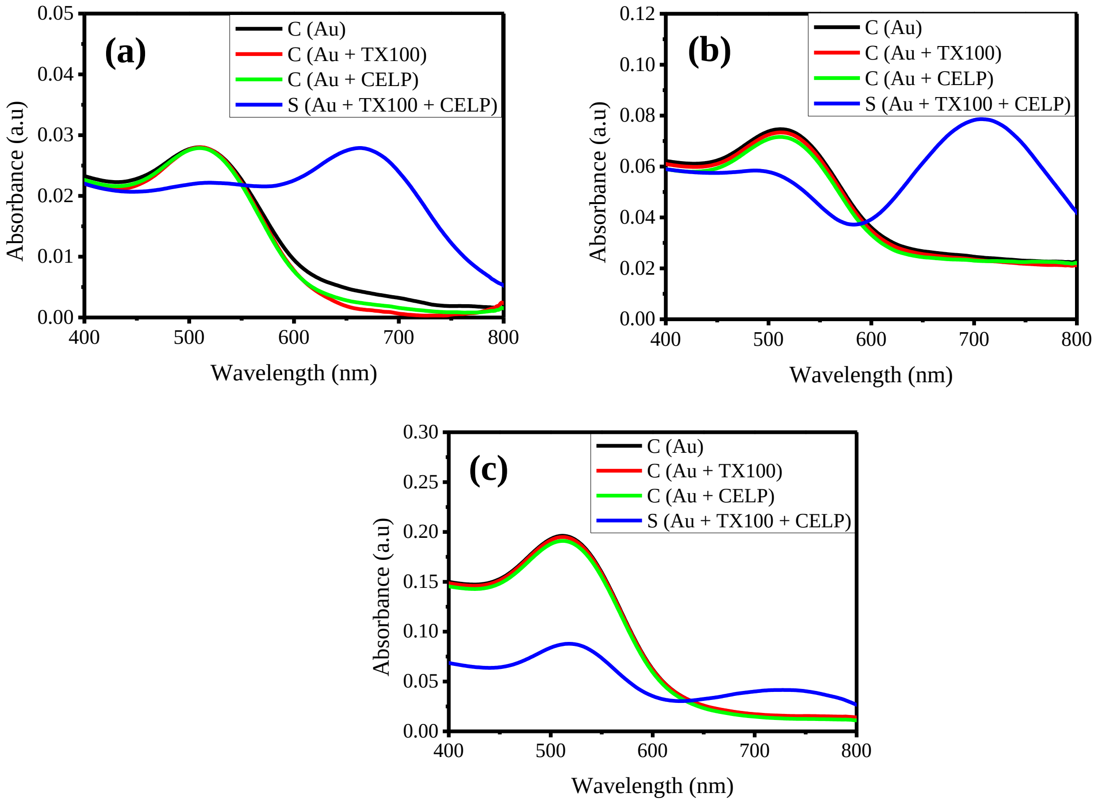

Figure S4. UV–vis spectra of (a) 0.06 mM, (b) 0.12 mM, and (c) 0.24 mM AuNPs: only AuNPs (**Au**), AuNPs with TX100 (**Au + TX100**), AuNPs with CELP (**Au + CELP**), and AuNPs with both TX100 and CELP (**Au + TX100 + CELP**).

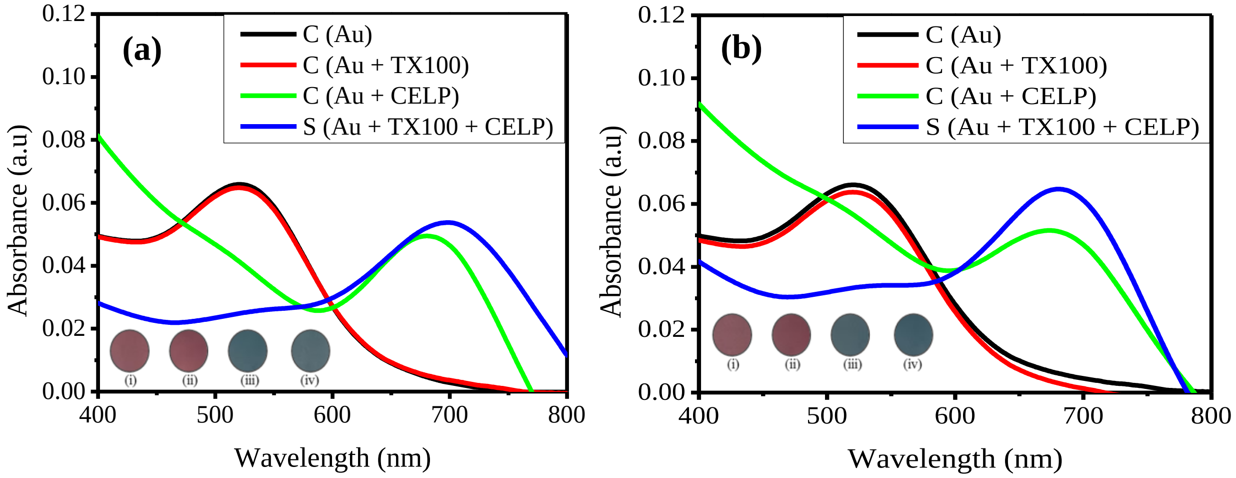

Figure S5. UV–vis spectra of 0.12 mM AuNPs in (a) SAB (pH 5.5) and (b) CPB (pH 5.0). Insets show the corresponding photographs of the AuNPs: only AuNPs (i); AuNPs with TX100 (ii); AuNPs with CELP (iii); AuNPs with TX100 and CELP (iv).

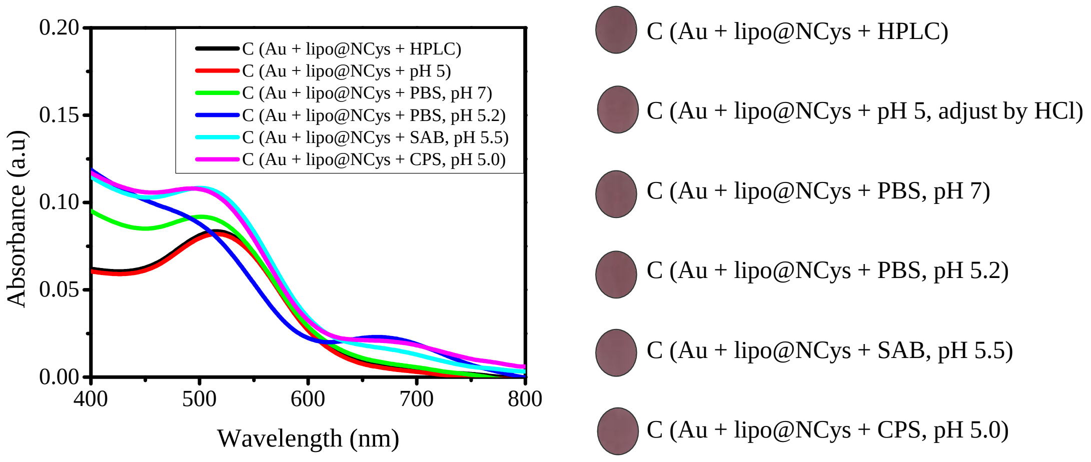

Figure S6. UV–vis spectra of AuNPs with liposomes that do not contain encapsulated Cys in various buffer solutions.
